# Attentional Bias of Individuals with Social Anxiety towards Facial and Somatic Emotional Cues in a Holistic Manner

**DOI:** 10.3390/bs14030244

**Published:** 2024-03-18

**Authors:** Yuetan Wang, Jingjing Liang, Ziwen Zhu, Jingyi Gao, Qiuyan Yao, Xiaobin Ding

**Affiliations:** School of Psychology, Northwest Normal University, Lanzhou 730030, China; 2021104099@nwnu.edu.cn (Y.W.); 2020220634@nwnu.edu.cn (J.L.); 2023220523@nwnu.edu.cn (Z.Z.); 2023220451@nwnu.edu.cn (J.G.); 2023220572@nwnu.edu.cn (Q.Y.)

**Keywords:** social anxiety, attentional bias towards facial and bodily emotions, integrative processing

## Abstract

Attentional bias towards threatening information is a crucial factor contributing to the development and persistence of social anxiety. However, the attentional bias towards threat information and the preferential processing pattern of emotional cues in individuals with social anxiety disorder during integrated facial and physical stimuli processing remain unclear. In this study, we employed a dot-probe paradigm to investigate the attentional bias towards integrated emotions (facial–body) among students with high and low levels of social anxiety (Experiment 1). Experiments 2 and 3 examined the attentional bias of socially anxious individuals when faced with conflicting emotional cues from faces or bodies in relation to integrated emotions. The data revealed that participants both high and low levels of social anxiety participants exhibited accelerated orienting and biased attention towards facial–body emotional processing. When there was inconsistency between emotional cues from faces or bodies and integrated emotions, higher levels of social anxiety were associated with increased vigilance towards threatening faces or bodies. These findings underscore that individuals with social anxiety possess an ability to rapidly capture threatening cues during the processing of facial–body emotional stimuli while also demonstrating a tendency to avoid relying solely on facial cues by compensating through bodily cues for emotion perception.

## 1. Introduction

Social anxiety (SA) refers to the negative emotional experience of individuals who fear receiving unfavorable evaluations from others in social situations. It is characterized by intense feelings of nervousness, unease, and distress, as well as a tendency to avoid social interactions. Social anxiety disorder or social phobia represents an extreme manifestation of social anxiety [1]. Importantly, in a survey report on the mental health status of Chinese college students in 2022, it was noted that 78.52% of students were not at risk for depression, while anxiety was more likely to be present in the lives of college students than depression, with only half of college students not at risk for anxiety (54.72%). Currently, social anxiety disorder ranks as the third most prevalent mental disorder after major depression and alcohol dependence, with a lifetime prevalence rate of 13.3%. Individuals with social anxiety disorder exhibit attentional bias towards threatening information in social contexts. This attentional bias towards threat plays a crucial role in perpetuating symptoms of social anxiety [2].

The attention bias towards threat information is a significant contributing factor to the persistence of social anxiety and may even be a causal factor in anxiety development [3]. Attention bias refers to the differential allocation of attention by individuals towards emotional or threatening stimuli compared to neutral stimuli [4]. In previous research, attention bias components primarily include facilitated attention or vigilance, difficulty in disengaging attention, and avoidance of certain stimuli [5]. The expression of threat information refers to directing some form of hostility towards the viewer, with anger being the most prominent example. Angry facial expressions can be perceived as aggressive to some extent and may imply disruption in social interactions; thus, angry faces represent a type of socially threatening stimulus. One perspective suggests that individuals with social anxiety maintain heightened alertness towards socially threatening stimuli such as angry faces, leading to increased attention and triggering an attention bias [6,7,8]. Another view is that socially anxious individuals develop attentional avoidance of negative emotions [9], which may serve as a safety behavior to reduce potential negative emotional experiences. Individuals with social anxiety particularly tend to avoid eye contact [9]. Based on the aforementioned perspectives, we will analyze the attention stage, which initially manifests as alertness and then transitions into avoidance [10]. Individuals with high levels of social anxiety exhibit noticeable attentional alertness to negative stimuli [11] and display tendencies towards avoidance [12]. The self-focused cognitive theory proposed by Wells et al. [13] suggests that socially anxious individuals tend to avoid directing their attention towards threatening cues in social situations, instead focusing inward and becoming trapped in previously constructed negative self-impressions, resulting in intense fear and anxiety. Additionally, Mogg et al. [14] first introduced the vigilance–avoidance hypothesis, suggesting that individuals with social anxiety disorder initially enter an automated phase of being captivated by socially threatening cues (initial hypervigilance), followed by an immediate shift into avoidance to reduce anxiety levels. Alternatively, individuals with social anxiety may struggle to disengage their attention from threat cues over time, leading to increased focus on these stimuli and difficulties in disengagement [15]. Extensive research has been conducted on both avoidance and vigilance of threat stimuli in facial stimulation regarding social anxiety disorders [16]. 

In studies of attentional bias in socially anxious individuals, a point-probing paradigm would be more sensitive to attentional bias for probing face stimuli or threatening stimuli [17]. Originating from Posner et al.’s [18] research on visual spatial attention and later formally proposed by Mathews et al. [19], the dot-probe paradigm has greatly advanced the study of attention biases. Research has shown that individuals with social anxiety exhibit accelerated attentional orientation towards threatening information and difficulties disengaging their attention when experimental stimuli are presented for less than 100 ms [20]. Cooper et al. [21] found that individuals did not show a negative bias towards angry faces when the negative stimulus was presented for 500 ms, whereas when the negative stimulus was presented for 100 ms, individuals showed an attentional bias towards negative information. Therefore, it can be inferred that the dot-probe paradigm measures initial orienting of attention towards stimuli during brief presentations, with stimulus presentation time being a significant factor influencing attention biases. Delchau et al. [3] used a modified dot-probe paradigm with emotional photographic faces to examine the effects of emotional cues on top-down control. Their results showed that people with higher levels of social anxiety showed greater attentional alertness to happy faces when they saw angry faces. However, this phenomenon was not observed when happy faces were paired with neutral ones. These results suggest that top-down control can effectively direct attention towards emotional faces; however, selective attention hypersensitivity for processing threatening emotional faces is specifically associated with heightened social anxiety [3]. Additionally, fearful and angry facial expressions elicit more pronounced attentional orienting compared to happy ones [22].

Additionally, in order to comprehend the emotions or intentions of others, individuals not only attend to their facial expressions but also their bodily movements [23,24]. When identifying emotions in naturalistic settings, body language is integrated with facial expressions. Due to the larger visual field and more expansive dynamic patterns of the body, anger may be more easily conveyed from a distance [25]. Body gestures and postures have become crucial cues for recognizing emotional states [26,27]. Moreover, Mariska’s research [28] revealed that individuals with high social anxiety tend to fixate on perceived objects’ bodies as a compensatory mechanism for the lack of emotional information resulting from avoiding faces. Importantly, during natural social interactions, our communication extends beyond mere facial expressions to encompass complete individuals [28]. Ding Xiaobin et al. [29] have demonstrated the striking similarity in cognitive processing mechanisms between bodily and facial expressions. It is evident that relying solely on facial expressions is inadequate for accurately perceiving others’ emotions; equal attention should be devoted to their entire body. Therefore, it is worthwhile to further investigate whether socially anxious individuals exhibit a preference for focusing on faces or bodies when confronted with congruent and incongruent face–body integration stimuli in images depicting overall emotional cues. However, in many cases, there will be “skin laughing meat not laughing” and “duplicity”, but the body movement is more difficult to control than the facial expression, resulting in emotional disharmony between the face and the body. Studies have shown that when face–body emotions are congruent, emotional processing will be promoted [30]. Then, when faces and body emotions are in conflict, the kind of emotional stimuli to which individuals with social anxiety will show an attention bias needs to be further explored.

The main aims of the study were to explore the attentional bias of socially anxious individuals in processing holistic emotional cues (face–body), to analyze in depth the attentional bias in the case of incongruence between the emotions of the face and the body, and to further summarize the effects of threatening information on attentional processing in socially anxious individuals. We hypothesized that when faces are emotionally congruent with the body, for integrated processing of emotions, participants with both high and low levels of social anxiety would have an attentional bias towards emotional stimuli as compared to neutral stimuli (Experiment 1); whereas when faces are emotionally incongruent with the body, for integrated processing of emotions, participants with high levels of social anxiety would have an attentional avoidance of angry faces (Experiment 2), participants with high levels of levels of social anxiety participants would be attentively alert to angry bodies (Experiment 3), and there would be accelerated attentional orienting as well as attentional disengagement difficulties.

## 2. Experiment 1: Characteristics of Face–Body Overall Emotional Cue Processing When the Stimulus Was Presented for 100 ms

### 2.1. Method 

Participants. In this study, Liebowitz Social Anxiety Scale (LSAS) was used to issue 536 questionnaires through an online platform to screen college students. In total, 123 invalid questionnaires were eliminated and 413 valid questionnaires were recovered. Finally, 216 people participated in this study. According to previous studies [31], participants scoring above 60 were identified as highly socially anxious (HSA, *n* = 108). Participants scoring below 35 were considered as having low social anxiety (LSA, *n* = 108). The cut-off score was reported to yield no false positive identification of SAD among non-SAD individuals [32]. To ensure that the screening procedure was successful, before the experimental session, participants completed the LSAS again and the Beck Depression Inventory (BDI) was used [33]. The sample size was determined using G Power 3.1 software as a reference and the analysis of variance (ANOVA) function was employed for repeated measures and interaction effects. Assuming α = 0.05 and 1− β = 0.95, with a minimum effect size f = 0.25 (medium effect), a recommended sample size of 28 was suggested. Experiment 1 collected data from 72 participants. Table 1 presents the demographic information of the three experiments and the results of *t*-tests for LSAS and BDI-II. All participants had no history or family history of mental illness; they had normal vision or corrected vision; all preferred right-handedness; none had previously participated in any research related to this experiment before; they provided informed consent prior to participating and received appropriate compensation upon completion. The study was approved by our local Ethics Committee.

Stimulus and apparatus. The facial emotion pictures were selected from the Chinese Facial Affective Picture System (CAFPS), which assessed the valence and arousal of emotional images. In total, 24 emotion faces (8 happy, angry, and neutral each) and 24 emotional body postures (8 happy, neutral, and angry each) were chosen as stimuli for this study. Previous studies have demonstrated that male anger expressions elicit stronger arousal responses in participants compared to female expressions of these emotions [34,35]. Faces and bodies were spliced into complete individuals based on the content of Experiment 1 (e.g., a happy face with a happy body spliced into an emotionally congruent and complete happy individual), and the experimental stimulus pictures were presented simultaneously with two complete individuals consisting of emotional stimulus pairs. There are four ways to combine them (angry–happy, angry–neutral, happy–neutral, neutral–neutral). The images have a gray background to eliminate irrelevant distractions. All face–body composite emotion clue images were processed in black and white using PhotoShop CS6 software while ensuring matching image size, brightness, and contrast. The dimensions of the images are 800 pixels by 1200 pixels with a resolution of 1024 × 768.

**Figure 1:**
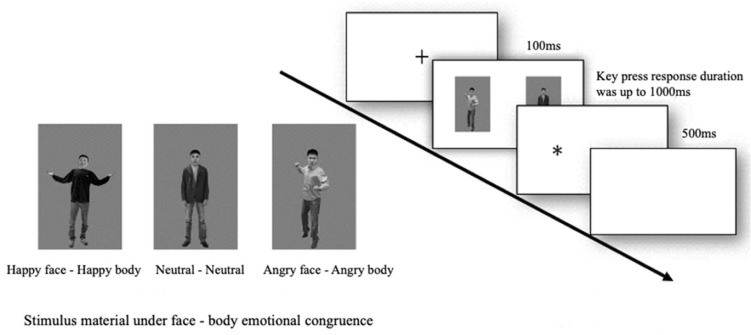
Flow chart of Experiment 1 (“+”: a fixation point, 100 ms; “*”: a probe, 500 ms).

Design and procedure. The experimental procedure was programmed using E-prime 2.0 software and was conducted in a quiet laboratory. Stimuli were presented on a liquid crystal display monitor with a resolution of 1920 × 1200. Viewing distance was set to 60 cm, and the entire experiment was conducted for approximately 5–6 min. First, a fixation point appears in the center of the screen (“+”, 100 ms), followed by a set of emotional stimulus pairs (i.e., two complete face–body emotional pictures are presented at the same time, 100 ms), and then a probe appears (“*”, 500 ms), and participants are required to make a judgment about the location of the probe on the screen and to press a key (if on the left side, the “D” key is pressed; if on the right side, the “K” key is pressed, 1000 ms); an empty screen is presented for 500 ms after the key is pressed. The left and right positions of the probe are balanced and randomly distributed. When happy/angry individuals and neutral individuals were presented simultaneously as a set of stimulus pairs, the presence of the probe on the side of the emotional individual was the congruent condition and the presence of the probe on the side of the neutral individual was the incongruent condition; when happy and angry individuals were presented simultaneously as a group, the presence of the probe on the side of the negative individual (i.e., angry) was the congruent condition, and the presence of the probe on the side of the positive individual (i.e., happy) was the incongruent condition. Emotion type combinations appear with equal probability and in random order. The four types of emotional facial expressions had equal probabilities throughout the experiment process and their order was randomized as depicted in Figure 1.

Data analysis. The data were analyzed using SPSS 22.0 for statistical purposes. Data points with reaction times below 200 ms and outliers exceeding three standard deviations were excluded as invalid, resulting in a final sample of 57 valid participants for statistical analysis. Among them, there were 33 participants in the high social anxiety group and 24 participants in the low social anxiety group. The error rates for all participants remained below 3%. Only trials on which participants correctly identified the probe were included in the RT analysis.

### 2.2. Results

RT. Three-factor repeated measurement ANOVA of 2 (subject type: HSA, LSA) × 3 (emotion pair type: A-H, A-N, H-N) × 2 (probe position: congruent, incongruent) was performed for response. The results showed that the main effect of the subject type was significant (F = 7.627, *p* = 0.008). The main effect of emotion on type was significant (F = 8.787, *p* < 0.01). The interaction between probe position and subject type was significant (F = 7.702, *p* = 0.007). Simple effect analysis showed that there were significant differences between HSA and LSA when the probe position was the same (*p* = 0.034). When the probe position is incongruent, there is a significant difference in the response time between HSA and LSA (*p* = 0.002). The interaction between probe position and emotion on type was close to significant (F = 3.761, *p* = 0.058). Simple effect analysis showed that when the probe position was the same, the difference between A-H and A-N mood pair types was significant (*p* < 0.01). There were significant differences between A-N and H-N emotional response types (*p* < 0.01). When the probe positions were incongruent, there was a significant difference in response time between A-H and A-N emotional pairs (*p* = 0.006). The descriptive statistics about the RT data of correctly judged by subjects in Experiment 1 are presented in Table 2. In addition, we conducted independent sample *t*-tests to compare the reaction times of HSA and LSA under three probe conditions (congruent, incongruent, neutral control). The results are shown in Table 3. There was no significant difference between HSA and LSA in the neutral baseline (N-N stimulus pair) control condition.

Attention component. (bias index, BI; orienting index, OI; disengaging index, DI). According to existing research, in the modified dot-probe paradigm, attention bias index (BI) refers to the discrepancy in reaction times (RTs) between incongruent and congruent conditions. A positive BI indicates heightened vigilance towards target stimuli, while a negative BI suggests avoidance of target stimuli. Facilitated attention (OI) is calculated by subtracting RTs for neutral conditions from congruent conditions. A positive score signifies accelerated attention towards target stimuli, whereas a negative score implies no facilitated attention towards target stimuli. Difficulty in attention disengagement (DI) is determined by subtracting RTs for incongruent conditions from neutral conditions. A positive score signifies challenges in disengaging attention from target stimuli, while a negative score suggests no difficulty in disengagement. Neutral–neutral emotional stimulus pairs were employed as control trials for baseline comparison and were included in the analysis solely for attention bias calculation. We conducted independent sample *t*-tests on the BI, OI, and DI of HSA and LSA. The results are shown in Table 3.

A three-factor repeated measures analysis of variance was conducted on attentional components, including two levels of participant type (HSA, LSA), three levels of emotion pair types (A-H, A-N, H-N), and three attentional components (BI, OI, DI). The results revealed that the interaction between participant type, emotion pair type, and attentional components was not statistically significant (F = 0.215, *p* = 0.929). However, there was a statistically significant interaction between participant type and attentional components (F = 3.320, *p* = 0.044), as well as a statistically significant interaction between emotion pair type and attentional components (F = 11.308, *p* < 0.001). Further analyses revealed that in terms of disengaging from difficult tasks, there was a significant difference between the high and low social anxiety groups (*p* = 0.022). In the condition where anger–happiness emotion pairs were presented, significant differences were found in both bias towards and orienting acceleration of attention (*p* = 0.040), as well as in bias towards and difficulty with disengagement of attention (*p* < 0.001). In the anger–neutral emotion condition, there was a significant difference observed in attentional bias acceleration and attentional disengagement difficulty (*p* = 0.043). In the happiness–neutral emotion condition, significant differences were found in attentional bias and attentional disengagement difficulty (*p* = 0.002), as well as in attentional orientation acceleration and attentional disengagement difficulty (*p* = 0.023). Regarding attentional bias, significant differences were identified among emotional types between anger–happiness and happiness–neutral conditions (*p* < 0.001), as well as between anger–happiness and anger–neutral conditions (0.025). Concerning attentional orientation acceleration, significant differences were observed among emotional types between anger–happiness and happiness–neutral conditions (*p* < 0.001). Finally, with respect to attentional disengagement difficulty, a significant difference was found among emotional types between anger–happiness and anger–neutral conditions (*p* = 0.006). To investigate the attentional bias towards threatening emotional stimuli of both groups, we performed independent sample *t*-tests on the attentional bias components for each type of emotional stimulus for HSA and LSA, as presented in Table 4.

### 2.3. Discussion

The findings from Experiment 1 suggest that individuals with high social anxiety demonstrate increased attentional avoidance towards threatening stimuli as the duration of presentation increases, compared to neutral stimuli. Conversely, individuals with low social anxiety transition from attentional vigilance to attentional avoidance. A stimulus presentation time of 100 ms allows for a more reliable observation of both high and low social anxiety individuals’ heightened attentional orientation towards threat stimuli and difficulty in disengaging their attention. These results are congruent with the “vigilance–avoidance” hypothesis proposed by Mogg et al. [14], which posits that individuals with social anxiety disorder initially exhibit automatic capture by socially threatening cues (referred to as initial vigilance stage) followed by immediate entry into an avoidance stage to alleviate their experience of anxiety. Furthermore, these findings align with Zhang et al.’s [36] meta-analysis description, suggesting that accelerated attentional orientation can be observed when cue presentation time is less than 100 ms or even subliminal, while attentional avoidance occurs during longer durations of threat stimulus presentation (typically greater than 1000 ms) and during late stages of cognitive processing.

Therefore, in the subsequent two studies, the stimulus presentation duration is maintained at 100 ms to facilitate a more comprehensive investigation into the constituents of attentional orienting facilitation and attentional disengagement difficulties. Martinez et al. [37] contend that both facial and bodily cues convey emotional information. While simultaneous processing of faces and bodies represents an optimal approach for recognizing basic emotions, individually, faces and bodies offer sufficient information to identify emotions above chance level. Hence, our objective is to independently examine the impact of facial and bodily stimuli on overall emotion processing.

## 3. Experiment 2: The Effect of Facial Emotion on the Processing of Face–Body Whole Emotional Cues

### 3.1. Method

Participants. We recruited 72 new participants in the same way as in Experiment 2, specifying that they had not taken part in the earlier experiment. Table 1 presents the demographic information for Experiment 2 and the results of *t*-tests conducted on LSAS and BDI-II scores.

**Figure 2:**
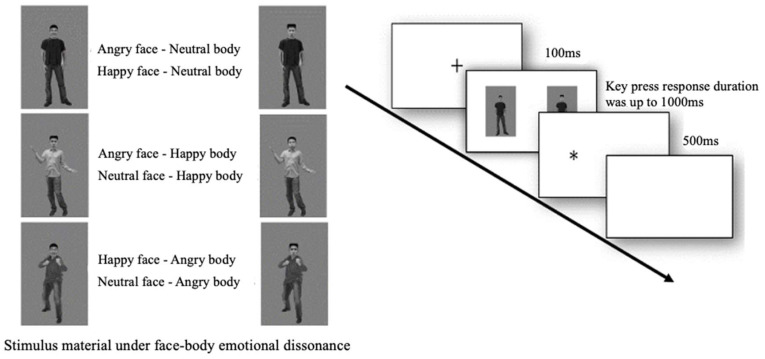
Flow chart of Experiment 2 (“+”: a fixation point, 100 ms; “*”: a probe, 500 ms).

Stimulus and apparatus. Faces and bodies were combined according to Experiment 2 to form stimulus pairs in which the emotions of faces and bodies were incongruent, and the experimental stimulus pictures were pairs of emotion stimuli consisting of two intact individuals presented simultaneously, differing from Experiment 1 in that the emotion stimulus pairs had a congruent somatic emotion and an incongruent type of emotion for the two faces (e.g., angry and happy faces were combined with two neutral bodies), and four combinations were made (32 in total, AH face–N body, AN face–H body, HN face–A body, and N face–N body). The experimental procedure remains congruent with Experiment 1, and identical equipment is utilized for presentation (see Figure 2).

Data analysis. In Experiment 2, the response time data of each subject that was incorrect, less than 200 ms, or outside plus or minus 3 standard deviations were excluded. A total of 59 subjects were included in the final statistical analysis, comprising 35 in the high social anxiety group and 24 in the low social anxiety group. It is worth noting that all subjects had an error rate lower than 3%.

### 3.2. Results

RT. The response time was analyzed using a three-factor repeated measures ANOVA, with subject type (HSA, LSA), emotion pair type (AH face–N body, AN face–H body, HN face–A body), and probe location (congruent, incongruent) as factors. The results revealed a significant main effect of probe location (F = 7.889, *p* = 0.007), participant type (F = 4.815, *p* = 0.032), and emotion–face pairing (F = 18.710, *p* < 0.001). There was also a significant interaction between probe location and participant type (F = 4.412, *p* = 0.040), probe location and emotion–face pairing (F = 12.809, *p* < 0.001), as well as emotion–face pairing and participant type (F = 3.668, *p* = 0.003). However, the interaction among probe location, emotion–face pairing, and participant type did not reach significance (F = 1.743, *p* = 0.182). The results of the simple effect analysis revealed significant differences in reaction time between the high and low social anxiety groups when the probe location remained congruent (*p* = 0.006). In the high social anxiety group, there were significant differences observed between stimuli featuring an angry face–neutral body combination and those featuring a neutral face–angry body combination (*p* < 0.001), as well as between stimuli featuring an angry face–neutral body combination and those featuring a happy face–neutral body combination (*p* = 0.001). In contrast, within the low social anxiety group, there were significant differences observed between stimuli featuring an angry face–neutral body combination and those featuring a neutral face–angry body combination (*p* = 0.005), as well as between stimuli featuring a neutral face–angry body combination and those featuring a happy face–neutral body combination (*p* = 0.001). The descriptive statistics of the RT data of correct judgment by subjects in Experiment 2 are presented in Table 5. In addition, we conducted independent sample *t*-tests to compare the reaction times of HSA and LSA under three probe conditions (congruent, incongruent, neutral control). The results are shown in Table 6. There was no significant difference between HSA and LSA in the neutral baseline (N-N stimulus pair) control condition.

Attention component. A three-factor repeated measures analysis of variance was conducted on the attentional components, examining two levels of participant type (HSA, LSA), three levels of emotion–face pairings (AH face–N body, AN face–H body, HN face–A body), and three attentional components (BI, OI, DI). Neutral–neutral emotional stimulus pairs were employed as control trials for baseline comparison and were included in the analysis solely for attention bias calculation. We conducted independent sample *t*-tests on the BI, OI, and DI of HSA and LSA. The results are shown in Table 6. The results revealed a significant interaction effect between participant type, emotion–face pairing type, and attentional components (F = 3.214, *p* = 0.024); as well as a significant interaction effect between emotion–face pairing type and attentional components (F = 17.326, *p* < 0.001). Simple effects analyses demonstrated that for the high social anxiety group in AH face–N body emotion–face pairings there were significant differences in attention biasing, attention-orienting facilitation, and attention disengagement difficulties (*p* < 0.001); all three components exhibited significant differences based on emotion–face pairing types (*p* = 0.008; *p* < 0.001; *p* < 0.001). For the low social anxiety group in AN face–H body emotion-face pairings there were significant differences in attention biasing, attention-orienting facilitation, and attention disengagement difficulties (*p* < 0.01); both attention biasing and orientation facilitation showed significant differences based on emotion–face pairing types (*p* = 0.002; *p* < 0.001). To investigate the attentional bias towards threatening emotional stimuli of both groups, we performed independent sample *t*-tests on the attentional bias components for each type of emotional stimulus for HSA and LSA, as presented in Table 7.

### 3.3. Discussion

The findings from Experiment 2 suggest that individuals with social anxiety display a distinct attention bias towards happy faces compared to neutral stimuli, which can be attributed to the impact of bodily postures on facial expression perception [34,38]. Previous studies have predominantly focused on utilizing facial stimuli, whereas this study incorporated bodily emotions. Kret et al. [28] proposed that individuals with high social anxiety exhibit heightened attention towards bodily expressions, particularly those conveying fear or anger. Consequently, the emotional processing of happy faces among individuals with social anxiety may be influenced by the presence of bodily emotions. In order to gain a deeper understanding of the impact of somatic emotions on integrated emotional processing, we introduced incongruent combinations of somatic emotions in stimulus pairs while maintaining congruent facial expressions in Experiment 3. This control experiment was conducted as a comparison to Experiment 2, where facial expressions were incongruent with integrated emotions. The objective was to further investigate how individuals with social anxiety perceive and process integrated emotions.

## 4. Experiment 3: The Effect of Somatic Emotion on the Processing of Face–Body Whole Emotional Cues

### 4.1. Method

Participants. We recruited 72 new participants in the same way as in Experiment 3, specifying that they had not taken part in the earlier experiment. Table 1 presents the demographic information for Experiment 3 and the results of *t*-tests conducted on LSAS and BDI-II scores.

**Figure 3:**
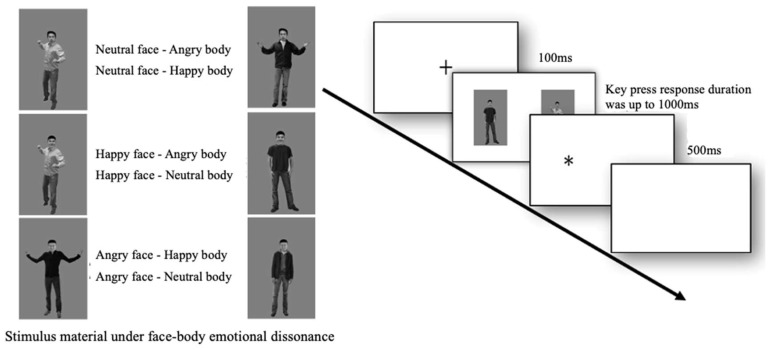
Flow chart of Experiment 3 (“+”: a fixation point, 100 ms; “*”: a probe, 500 ms).

Stimulus and procedure. The stimuli were identical to those used in Experiment 1, displayed using the same equipment. Faces and bodies were combined to compose stimulus pairs with incongruent face and body emotions. The experimental stimulus pictures were emotion stimulus pairs consisting of two complete individuals presented at the same time, differing from Experiment 2 in that the body emotions in the emotion stimulus pairs were congruent, the emotion types of the two faces were incongruent (e.g., angry and happy bodies were combined with two neutral faces), and there were four ways of combining the pairs (32 in total, A face–HN body, N face–AH body, H face–AN body, and N face–N body) (see Figure 3).

Data analysis. In Experiment 3, data from participants with incorrect responses, reaction times less than 200 ms, and outliers beyond three standard deviations were excluded. A total of 55 valid participants (33 in the high social anxiety group and 22 in the low social anxiety group) were included for statistical analysis. The error rates for all participants remained below 3%.

### 4.2. Result

RT. A repeated measures analysis of variance with three factors was conducted to examine reaction time for tasks involving two participant types (HSA, LSA), three emotion–face pairings (A face–HN body, H face–AN body, N face–AH body), and two probe positions (congruent and incongruent). The results revealed a significant main effect for emotion–face pairings (F = 16.112, *p* < 0.001). There was also a significant interaction between probe position and participant type (F = 5.956, *p* = 0.018), as well as a significant interaction between probe position and emotion–face pairings (F = 4.017, *p* = 0.024). Further analysis showed that the low social anxiety group exhibited significantly different reaction times between congruent and incongruent probe positions (*p* = 0.037). Specifically, under congruent probe conditions, there was a significant difference in reaction time between the A face–HN body pairing and H face–AN body pairing (*p* < 0.001). Conversely, when the probes were incongruent, there was a significant difference in reaction time between the A face–HN body pairing and N face–AH body pairing (*p* = 0.005). No significant interaction effect was observed between emotion–face pairings and participant types (F = 0.877, *p* = 0.163); nor among the interactions of probe with both emotion–face pairings and participant types (F = 0.913, *p* = 0.158). The descriptive statistics of the RT data of correct judgment by subjects in Experiment 3 are presented in Table 8. In addition, we conducted independent sample *t*-tests to compare the reaction times of HSA and LSA under three probe conditions (congruent, incongruent, neutral control). The results are shown in Table 9. There was no significant difference between HSA and LSA in the neutral baseline (N-N stimulus pair) control condition.

Attention component. A three-factor repeated measures analysis of variance was conducted on attentional components, examining two levels of participant type (HSA, LSA), three levels of emotion–face pairings (A face–HN body, H face–AN body, N face–AH body), and two levels of probe location (congruent and incongruent). Neutral–neutral emotional stimulus pairs were employed as control trials for baseline comparison and were included in the analysis solely for attention bias calculation. We conducted independent sample *t*-tests on the BI, OI, and DI of HSA and LSA. The results are shown in Table 9. The results indicated that there was no significant interaction among participant type, emotion–face pairing type, and attentional components (F = 1.645, *p* = 0.178). However, a significant interaction was found between participant type and attentional components (F = 3.325, *p* = 0.044), as well as between emotion–face pairing type and attentional components (F = 17.326, *p* < 0.001). Further analyses revealed significant differences in attention bias between the high social anxiety group and the low social anxiety group (*p* = 0.018). Specifically within the low social anxiety group, there were significant differences in both attention bias and difficulty disengaging from attention (*p* = 0.005), as well as in accelerated orienting of attention compared to difficulty disengaging from attention (*p* = 0.033). Moreover, in the condition where anger–happy facial expressions were paired with neutral bodies, all three types of attentional components showed significant differences (*p* < 0.01). To investigate the attentional bias towards threatening emotional stimuli of both groups, we performed independent sample *t*-tests on the attentional bias components for each type of emotional stimulus for HSA and LSA, as presented in Table 10.

### 4.3. Discussion

The findings from Experiment 2 and Experiment 3 further validate that individuals with social anxiety exhibit a heightened attentional focus on bodily emotions rather than facial emotions, compensating for the loss of emotional information resulting from face avoidance. The observed differential attentional bias towards angry bodies under different presentation conditions suggests a bidirectional contextual effect between faces and bodies [20], indicating that facial emotions also influence bodily emotions. Therefore, in Experiment 3, when there is an inconsistency between facial and bodily emotions and the face remains neutral, it effectively reveals the attentional bias towards bodily emotions. This outcome supports our hypothesis that socially anxious individuals tend to avoid faces in their daily lives while relying on bodily emotions as a compensatory strategy. Kret et al. [28] discovered that individuals with high social anxiety, compared to those with low social anxiety, demonstrate increased fixation on perceptual body expressions as a means of compensating for the loss of emotional information caused by avoiding faces. One possible explanation for this phenomenon is that anger differs from other emotions because it represents a direct or imminent threat to the observer, triggering heightened startle reflexes and fight-or-flight responses [39].

## 5. General Discussion

The present study utilized a modified dot-probe paradigm to examine the attentional bias towards facial–body emotional cues in individuals with social anxiety. It has been discovered that congruency between facial and bodily emotions facilitates emotion processing, while incongruency impedes it [20]. When stimuli were presented for a duration of 100 ms, individuals with higher levels of social anxiety demonstrated a shift from vigilance to avoidance in their attentional bias, as well as a transition from difficult-to-disengage attention to easy-to-disengage attention. Both highly socially anxious individuals and those with low levels exhibited accelerated attentional orientation towards emotional stimuli, enabling them to rapidly capture such emotional cues. This finding is congruent with previous research conducted by Gilboa et al. [40], who investigated the attentional bias of anxious individuals using a visual search paradigm and observed that compared to non-anxious individuals, anxious individuals displayed reduced reaction time towards angry faces than towards happy faces when neutral faces served as baseline stimuli. This suggests that anxious individuals are capable of swiftly detecting threatening information [40]. With regard to the attentional bias towards angry face–body emotions containing threat-related information, due to the presentation duration of 100 ms, participants quickly developed alertness in their attention. This result aligns with Mogg et al.’s [14] “vigilance–avoidance” hypothesis cognitive model for social anxiety disorder which proposes that socially anxious individuals initially enter an initial alert stage where they are automatically captured by social threat-related cues before promptly transitioning into an avoidance stage aimed at reducing anxiety experience.

When presented with both emotional and neutral stimuli, individuals with high social anxiety tend to avoid attending to angry emotions, particularly focusing on bodily expressions rather than facial expressions. However, they exhibit heightened vigilance towards happy faces while avoiding attending to happy body movements. One possible explanation is that the exuberant movements associated with happiness can be perceived as threatening by individuals with high social anxiety, thus leading to increased vigilance towards bodies. Research has shown a bidirectional contextual effect between facial and bodily emotion recognition [41], indicating that the recognition of facial emotions can be influenced by bodily emotions. Therefore, the heightened vigilance towards happy faces may be attributed to the influence of anger in bodily expressions within their emotional combination.

Individuals diagnosed with social anxiety disorder are more inclined to avoid observing others’ facial expressions during social interactions in comparison to individuals without anxiety [42]. This avoidance behavior may be driven by a high level of self-focus in social interactions, as individuals with social anxiety tend to anticipate negative evaluations from others [43]. Research conducted by Wenzler et al. [44] on facial–body emotion suggests that participants maintain probabilistic levels of valence and arousal for isolated facial emotions but accurately perceive emotional valence based on bodily emotions and holistic cues from face–body emotion stimuli. Therefore, when there is ambiguity in recognizing facial emotions, bodily emotions effectively serve as compensatory signals [20].

This research is not without limitations. First, we hope that future research will replicate and extend our findings, for instance, by using larger samples and/or participants with (sub)clinical levels of anxiety to enhance the understanding of the interplay between temporary goals and anxiety in attentional bias to threat. Secondly, future research could adopt more reliable means (i.e., behavioral tasks, ERP, fMRI) to measure attention control and to further explore the relationship among the variables with a more reasonable sample. Thirdly, this study only used angry facial expressions as threat stimuli, and the number of experimental trials was not sufficient. Future research could explore whether there are differences in attention bias towards different threatening faces (such as disgusted and fearful expressions) among individuals with social anxiety and increase the number of trials. Finally, due to differences in cultural backgrounds, sometimes people express threats with slightly different facial expressions and body movements, which can also have an impact on the results of studies, for example, in some cultures, a smiling/laughing face may signal a social threat; the raising of hands in the West when expressing happiness might be considered as a warning of aggression in Chinese culture, so the discussion of cross-cultural contexts could be added to future research on threatening emotions.

## 6. Conclusions

Research findings indicate that individuals with social anxiety initially display an attentional bias towards emotional stimuli, which subsequently transitions from vigilance to avoidance as the duration of stimulus presentation increases in order to alleviate anxiety. This aligns with the “vigilance–avoidance” hypothesis regarding attentional bias in social anxiety. When processing facial–body emotion cues holistically, socially anxious individuals tend to avoid focusing on facial stimuli and instead rely more on bodily cues for emotional perception.

## Figures and Tables

**Table 1:** Differences between high and low social anxiety groups on the LSAS and the BDI-II depression scale.

Group	Age	LSAS	BDI-II
	*M ± SD*	*M ± SD*	*M ± SD*
Experiment 1			
HSA (*n =* 33)	24.12 ± 2.421	80.30 ± 18.684	3.45 ± 3.800
LSA (*n =* 24)	23.96 ± 2.710	18.21 ±10.579	1.46 ± 2.284
*t*	0.238	15.905 ***	2.467 *
Experiment 2			
HSA (*n =* 35)	24.57 ± 2.913	79.37 ± 18.916	3.69 ± 3.652
LSA (*n =* 24)	23.79 ± 2.750	18.58 ± 9.740	1.29 ± 1.829
*t*	1.033	16.145 ***	3.318 **
Experiment 3			
HSA (*n =* 33)	24.58 ± 3.011	79.21 ± 19.390	16.95 ± 11.337
LSA (*n =* 22)	24.00 ± 2.619	3.70 ± 3.695	1.45 ± 2.365
*t*	0.731	14.996 ***	2.519 *

Note. LSAS represents the Liebowitz Social Anxiety Scale, BDI-II represents the Beck Depression Inventory, Second Edition, HSA represents the high social anxiety group and LSA represents the low social anxiety group. ***: *p* < 0.001, **: *p* < 0.01, *: *p* < 0.05, the same as below.

**Table 2:** Reaction time data for face–body emotional pairs in high and low social anxiety groups (Experiment 1).

Emotional Pair Type	Location of Stimulus Presentation	High Social Anxiety Group	Low Social Anxiety Group
		*M ± SD* (*n* = 33)	*M ± SD* (*n* = 24)
Anger–Happiness	Conformity	328.138 ± 34.674	348.436 ± 37.999
	Inconformity	338.097 ± 39.102	370.640 ± 54.509
Anger–Neutral	Conformity	331.769 ± 39.304	351.822 ± 51.625
	Inconformity	325.115 ± 25.723	357.771 ± 46.658
Happy–Neutral	Conformity	345.919 ± 37.488	371.581 ± 52.910
	Inconformity	333.866 ± 37.817	363.621 ± 45.804
Neutral–Neutral		336.234 ± 29.768	357.423 ± 44.243

**Table 3:** The reaction time data of the high and low social anxiety groups under different probe conditions and the attention bias component data of the high and low social anxiety groups at 100 ms of stimulus presentation time (Experiment 1).

	High Social Anxiety Group	Low Social Anxiety Group		
	*M ± SD* (*n* = 33)	*M ± SD* (*n* = 24)	*t*	*p*
Reaction times under different probe conditions				
Probe point agreement	335.300 ± 33.491	357.117 ± 42.559	−2.166 *	0.035 *
Probe point without agreement	332.149 ± 29.96	364.106 ± 45.087	−3.021 **	0.005 **
Neutral control	336.234 ± 29.768	357.423 ± 44.243	−2.035 *	0.051
Attentional bias component				
BI	−3.150 ± 17.727	6.989 ± 19.494	−2.045 *	0.046 *
OI	0.934 ± 17.658	0.306 ± 21.737	0.120	0.905
DI	−4.085 ± 15.343	6.682 ± 17.708	−2.451 *	0.017 *

Note. BI represents attentional bias, OI represents attentional orienting acceleration, and DI represents attentional disengagement difficulties. **: *p* < 0.01, *: *p* < 0.05, the same as below.

**Table 4:** Attentional bias component data for each emotion pair type for the high and low social anxiety groups (Experiment 1).

Emotional Pair Type	Variable	High Social Anxiety Group	Low Social Anxiety Group		
		*M* ± *SD* (*n =* 33)	*M* ± *SD* (*n =* 24)	*t*	*p*
Anger–Happiness	BI	9.959 ± 29.180	22.204 ± 37.256	−1.391	0.170
	OI	8.096 ± 18.356	8.987 ± 28.810	−0.142	0.887
	DI	1.863 ± 26.976	13.217 ± 26.377	−1.584	0.119
Anger–Neutral	BI	−6.654 ± 35.914	5.949 ± 35.994	−1.307	0.197
	OI	4.465 ± 28.109	5.601 ± 32.718	−0.140	0.889
	DI	−11.119 ± 19.506	0.348 ± 23.074	−2.029 *	0.047 *
Happy–Neutral	BI	−12.053 ± 25.104	−7.96 ± 35.394	−0.511	0.611
	OI	−9.685 ± 24.368	−14.158 ± 31.030	0.610	0.545
	DI	−2.368 ± 23.251	6.198 ± 29.103	−1.235	0.222

Note. BI represents attentional bias, OI represents attentional orienting acceleration, and DI represents attentional disengagement difficulties. *: *p* < 0.05.

**Table 5:** Reaction time data for face–body emotional pairs in high and low social anxiety groups (Experiment 2).

Emotional Pair Type	Location of Stimulus Presentation	High Social Anxiety Group	Low Social Anxiety Group
		*M* ± *SD* (*n =* 35)	*M* ± *SD* (*n =* 24)
AH face–N body	Conformity	322.051 ± 31.187	349.503 ± 40.184
	Inconformity	314.695 ± 36.000	338.549 ± 38.520
AN face–H body	Conformity	342.911 ± 33.319	371.437 ± 41.613
	Inconformity	333.679 ± 41.714	339.980 ± 34.041
HN face–A body	Conformity	325.398 ± 34.867	342.124 ± 33.040
	Inconformity	335.851 ± 41.600	342.002 ± 32.204
Neutral–Neutral		333.528 ± 34.769	349.528 ± 34.219

Note. A: angry, H: happy, N: neutral. AH face–N body represents the same neutral body combined with angry and happy faces, respectively, AN face–H body represents the same happy body combined with angry and neutral faces, respectively, HN face–A body represents the same angry body combined with happy and neutral faces, respectively.

**Table 6:** The reaction time data of the high and low social anxiety groups under different probe conditions and the attention bias component data of the high and low social anxiety groups at 100 ms of stimulus presentation time (Experiment 2).

	High Social Anxiety Group	Low Social Anxiety Group		
	*M* ± *SD* (*n =* 35)	*M* ± *SD* (*n =* 22)	*t*	*p*
Reaction times under different probe conditions				
Probe point agreement	330.300 ± 30.341	353.978 ± 33.634	−2.817 **	0.007 **
Probe point without agreement	327.962 ± 36.932	340.127 ± 30.273	−1.334	0.187
Neutral control	333.247 ± 34.769	349.528 ± 34.219	−1.778	0.081
Attentional bias component				
BI	−2.337 ± 19.098	−13.850 ± 24.635	2.020 *	0.048 *
OI	2.947 ± 14.310	−4.449 ± 23.635	1.373	0.178
DI	−5.284 ± 18.024	−9.400 ± 19.358	0.836	0.407

Note. BI represents attentional bias, OI represents attentional orienting acceleration, and DI represents attentional disengagement difficulties. **: *p* < 0.01, *: *p* < 0.05, the same as below.

**Table 7:** Attentional bias component data for each emotional stimulus pair for the high and low social anxiety groups (Experiment 2).

Emotional Pair Type	Variable	High Social Anxiety Group	Low Social Anxiety Group		
		*M* ± *SD* (*n =* 35)	*M* ± *SD* (*n* = 24)	*t*	*p*
Angry face–Neutral body Happy face–Neutral body	BI	−7.356 ± 23.444	−10.954 ± 31.522	0.503	0.617
	OI	11.477 ± 19.917	0.025 ± 32.692	1.495	0.144
	DI	−18.833 ± 22.524	−10.979 ± 23.855	−1.239	0.221
Angry face–Happy body Neutral face–Happy body	BI	−9.232 ± 26.593	−31.457 ± 37.983	2.647 **	0.010 **
	OI	−9.383 ± 19.254	−21.909 ± 31.836	1.685	0.101
	DI	0.431 ± 22.995	−9.548 ± 27.319	1.516	0.135
Happy face–Angry body Neutral face–Angry body	BI	10.453 ± 33.726	−0.122 ± 34.038	1.179	0.243
	OI	7.848 ± 18.307	7.404 ± 27.966	0.074	0.941
	DI	2.603 ± 25.379	−7.526 ± 26.935	1.469	0.147

Note. BI represents attentional bias, OI represents attentional orienting acceleration, and DI represents attentional disengagement difficulties. **: *p* < 0.01.

**Table 8:** Reaction time data for face–body emotional pairs in high and low social anxiety groups (Experiment 3).

Emotional Pair Type	Location of Stimulus Presentation	High Social Anxiety Group	Low Social Anxiety Group
		*M* ± *SD* (*n =* 33)	*M* ± *SD* (*n =* 22)
A face–HN body	Conformity	321.398 ± 33.379	333.547 ± 39.256
	Inconformity	317.799 ± 43.753	351.265 ± 41.25
H face–AN body	Conformity	346.218 ± 34.973	348.715 ± 45.269
	Inconformity	330.399 ± 35.921	351.151 ± 33.586
N face–AH body	Conformity	331.748 ± 37.624	349.277 ± 43.587
	Inconformity	338.758 ± 37.296	355.580 ± 38.267
Neutral–Neutral		334.973 ± 38.445	356.705 ± 39.840

Note. A: angry, H: happy, N: neutral. A face–HN body represents the emotional pair of the same angry face combined with happy and neutral body, respectively; H face–AN body represents the emotional pair of the same happy face combined with angry and neutral body, respectively; N face–AH body represents the emotional pair of the same neutral face combined with angry and happy face, respectively.

**Table 9:** The reaction time data of the high and low social anxiety groups under different probe conditions and the attention bias component data of the high and low social anxiety groups at 100 ms of stimulus presentation time (Experiment 3).

	High Social Anxiety Group	Low Social Anxiety Group		
	*M* ± *SD* (*n =* 33)	*M* ± *SD* (*n =* 22)	*t*	*p*
Reaction times under different probe conditions				
Probe point agreement	333.493 ± 30.824	343.917 ± 36.752	−1.137	0.261
Probe point without agreement	329.252 ± 33.576	352.447 ± 33.892	−2.500 *	0.016 *
Neutral control	334.973 ± 38.445	356.705 ± 39.840	−2.024 *	0.051
Attentional bias component				
BI	−4.240 ± 18.050	8.529 ± 20.628	−2.427 *	0.019 *
OI	1.479 ± 17.990	12.787 ± 23.945	−1.999	0.051
DI	−5.720 ± 14.580	−4.258 ± 26.785	−0.234	0.817

Note. BI represents attentional bias, OI represents attentional orienting acceleration, and DI represents attentional disengagement difficulties. *: *p* < 0.05, the same as below.

**Table 10:** Attentional bias component data for each emotion pair type for the high and low social anxiety groups (Experiment 3).

Emotional Pair Type	Variable	High Social Anxiety Group	Low Social Anxiety Group		
		*M* ± *SD* (*n =* 33)	*M* ± *SD* (*n =* 22)	*t*	*p*
Angry face–Happy body Angry face–Neutral body	BI	−3.599 ± 42.263	17.718 ± 37.512	−1.915	0.061
	OI	13.575 ± 27.657	23.158 ± 34.551	−1.139	0.260
	DI	−17.174 ± 25.046	−5.44 ± 32.732	−1.504	0.138
Happy face–Angry body Happy face–Neutral body	BI	−15.819 ± 28.750	2.436 ± 31.315	−2.226 *	0.030 *
	OI	−11.245 ± 23.809	7.99 ± 30.382	−2.626 *	0.011 *
	DI	−4.574 ± 24.915	−5.554 ± 28.606	0.135	0.893
Neutral face–Angry body Neutral face–Happy body	BI	7.01 ± 28.915	6.303 ± 33.373	0.084	0.933
	OI	3.225 ± 21.789	7.428 ± 32.474	-0.575	0.568
	DI	3.785 ± 20.967	−1.125 ± 34.671	0.655	0.515

Note. BI represents attentional bias, OI represents attentional orienting acceleration, and DI represents attentional disengagement difficulties. *: *p* < 0.05.

## Data Availability

The raw data supporting the conclusions of this article will be made available by the authors on request.
